# Experimental Evaluation of Sodium Silicate-Based Nanosilica against Chloride Effects in Offshore Concrete

**DOI:** 10.1155/2014/541035

**Published:** 2014-12-10

**Authors:** Kyoung-Min Kim, Hak-Young Kim, Young-Sun Heo, Sang-Jin Jung

**Affiliations:** ^1^Architectural Technology Research Team, Daewoo Institute of Construction Technology, Suwon 440-800, Republic of Korea; ^2^Department of Architectural Engineering, Dankook University, Yongin 448-701, Republic of Korea; ^3^Fire Research Center, Korea Institute of Civil Engineering and Building Technology (KICT), Hwaseong 445-861, Republic of Korea

## Abstract

This study investigates the effect of a new pore filling material, named sodium silicate-based nanosilica (SS), on resisting the diffusion of the chloride ions. The proposed SS is chosen, mainly due to its smaller particle size, compared to the conventional ethyl silicate-based nanosilica. Each particle of SS is chemically treated to have the negative (−) charge on its surface. Four types of mixes with different amounts of partial replacement with fly ash and slag are prepared. Effect of water to binder ratios (0.35, 0.40, and 0.45) is also examined. Test results showed that the inclusion of SS was significantly beneficial for protecting the concrete from chloride attack. At a given strength, the SS inclusion in concrete was up to three times more effective than the control concrete without SS. It is believed that these excellent results are attributed to the small particle size and the chemical surface treatment of SS. In this study, experiments of compressive strength, hydration heat, accelerated neutralization, and sulfate erosion tests were also conducted to find the general effect of SS inclusion on the fundamental properties and durability of concrete.

## 1. Introduction

The reinforced concrete that is used in structures constructed in coastal resorts and areas, which are likely to increase in number due to increases in maritime trade and city construction on coastal landfills, is impacted by chloride-induced corrosion by chlorine ions. Corrosion damage to such structures is often visible in rust staining of the surface cracking and detachment of the concrete cover due to the corrosion and expansion of reinforcement bars [1, 2]. Rust decreases the cross-sectional area of the reinforced concrete and thus reduces the adhesive power of the steel reinforcements and the cover concrete as the polymer generated by chloride ions. As a result, chloride-induced corrosion is a major cause of reduction in the durability life of structures built in coastal areas [3, 4].

The cause of the presence of the chloride ions in concrete takes place in two ways: one is from the pure components of concrete, such as water, aggregate, and cement, and the other is the intrusion of the chloride ions from atmosphere. In the case of structures that are exposed to marine environments, the chloride ions directly penetrate from the sea water or by sea salt particles, whereas in nonmarine environments, penetration caused by winter deicing salt is the primary path [5, 6]. The majority of cases in which there is impact on the durability life of reinforced concrete involve chloride intrusion from the external environment [7]. Nagataki et al. found that the risk of chloride penetration from the outside is two or three times higher than that from internal chloride. This is due to the formation of Friedel's salt that the chloride anions in cementitious composites react with monosulfate (C_3_A*·*3CaSO_4_ *·*12H_2_O) and replace sulfate ions. The other reason is that, after concrete is cured, the chloride penetration from the outside is hard to resolve [8, 9].

In general, measures designed to suppress corrosion due to salt damage in reinforced concrete structures can be divided into three types. The first one is the use of a primary barrier by the coating of the concrete surface. Secondly, barriers may be used in which the chloride ions are bound by the physical method of increasing the density of the concrete itself and the insoluble chloride. Finally, the third type of barrier involves the formation of a passive film on the surface of the rebar.

In this paper, the second type of barriers is adopted. In previous research work conducted by authors [10], a new nanosized pore-filling material, named sodium silicate-based nanosilica, was first introduced. In this previous work, the effect of the proposed sodium silicate-based nanosilica (SS) on pore structures of cement pastes was investigated, compared to that of the conventional ethyl silicate-based nanosilica (ES). It was found that the inclusion of SS in cement pastes was much more beneficial for producing dense pore structures. However, this conclusion was based on the results of samples with a cement paste condition. Hence, in the current study, SS was applied to the samples with a concrete condition, and then this study further investigated the effect of SS inclusion on the properties of concrete especially in marine environment, where the chloride attack occurs. To upgrade the effect of SS on the marine environment, the induction of electrochemical equilibrium of chloride ions through the negative charge formation on silica surface was proceeded.

## 2. Sodium Silicate-Based Nanosilica (SS)

### 2.1. Development and Production

Various mineral and chemical admixtures are able to improve the performance of concrete and to meet the design requirements for this type of work [11, 12]. Among such admixtures, nanosilica is a particularly important material that significantly contributes to rapid hydration and capillary pore-filling due to its small particle size and high specific surface area [13]. It can thus meet the performance requirement that cover concrete should be dense enough to prevent or reduce the intrusion of sea salt particles and sea water.

Sodium silicate-based nanosilica (SS) is introduced in previous work [10]. This was developed to be suitable for use in offshore construction concrete and represents an improvement on the physical and chemical characteristics, compared to the conventional ethyl silicate-based nanosilica (ES) [14].

**Figure 1:**
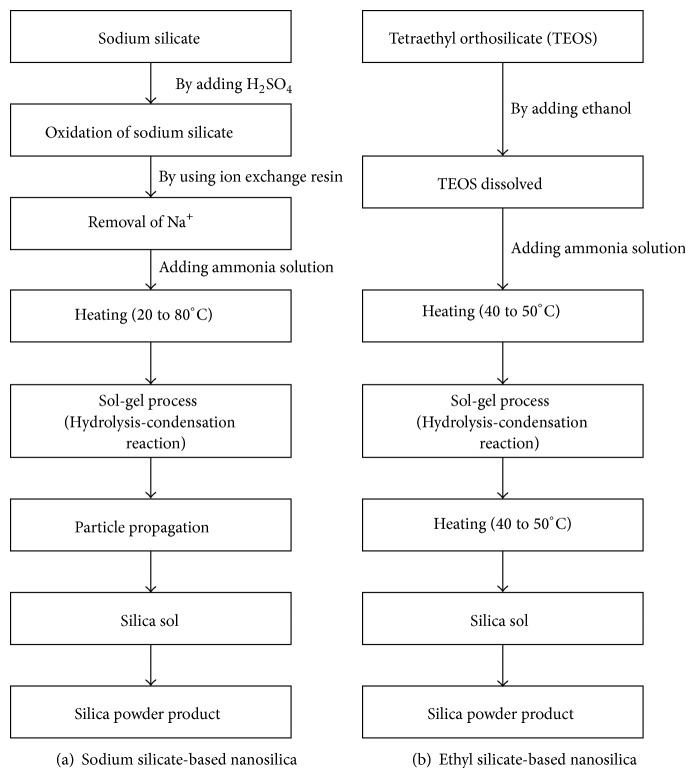
Synthesis process for nanosilica products.

Final product of SS is in powder form with surface coating with membrane so that the organic oligomer was polymerized.  Figure 1 shows the order of the SS production process in comparison with that for the conventional ES [10].

A sol-gel process involving hydrolysis and condensation using tetraethyl orthosilicate (TEOS) is widely used to synthesize nanosilica. A number of researchers [15, 16] have hypothesized that the concentration of TEOS, the ratio of water to TEOS, the feed rate of ammonia, the concentration of solvent, and the reaction temperature are the five critical parameters that control the nucleation, growth, size, and specific surface area of the nanosilica.

**Figure 2:**
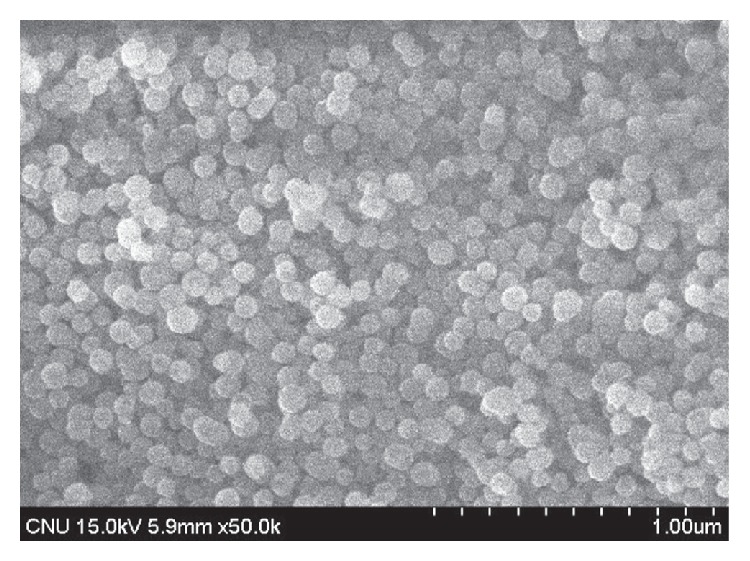
SEM micrograph of sodium silicate-based nanosilica (×50 k).

**Figure 3:**
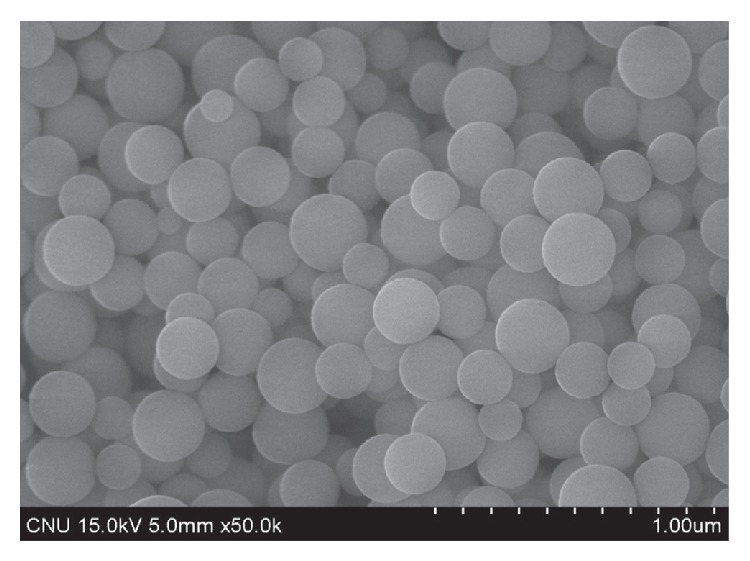
SEM micrograph of ethyl silicate-based nanosilica (×50 k).

However, nanosilica cement based on ethyl silicates such as TEOS can cause many weaknesses in the application of cement composites. Firstly, the particle size of ES is about 3 times greater than that of SS (Figures 2 and 3). This element is also very constrained in the production of more effective admixture material. Secondly, the cost of sodium silicate is approximately four times less than that of ethyl silicate in Korea, due to its lower purity, and it is expected to be reproducible with a small particle size.

### 2.2. Properties as Related to Off-Shore Structures

**Figure 4:**
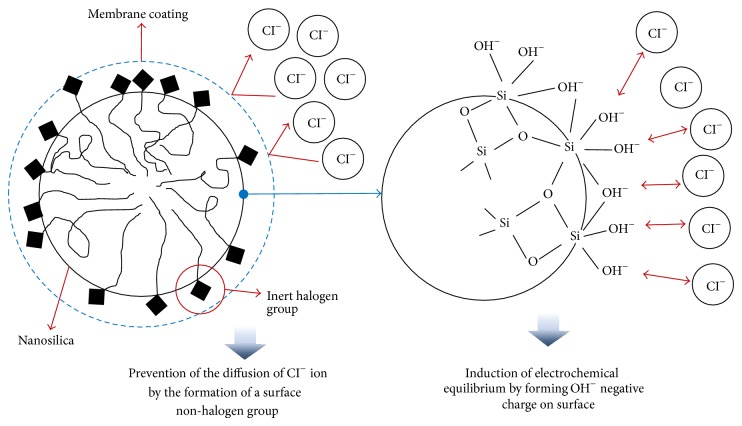
Conceptual diagram for chemical treatment on the surface of SS.

If SS is applied to the concrete in a marine structure, it affects this concrete through the following three actions. The first action is quick pozzolanic reaction, and the second one is the pore-filling effect within the cement matrix. These actions physically create the dense concrete and prevent the intrusion of sea water and sea salt particles. The third action is to chemically prevent the diffusion of Cl (−) ions while inducing electrochemical equilibrium through the negative (−) charge formation of the nanosilica surface and the inert halogen in the same group as the internal Cl (−) ions, as shown in Figure 4.

**Figure 5:**
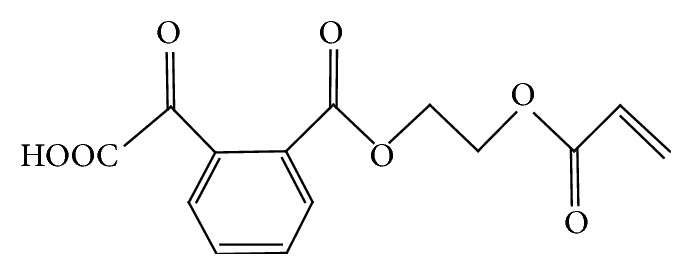
Chemical structure of half-esterification reaction product.

The inert halogen group formed on the surface is made by synthesis of the nanosilica and organic oligomers. For synthesis of the organic oligomers, denatured halogenated epoxy acrylate was synthesized through the half-esterification reaction from anhydride or halogenated anhydride.  Figure 5 shows the chemical structure of the half-esterification reaction product.

## 3. Experiment

### 3.1. Experimental Procedure

In this section, various engineering properties are reviewed in order to evaluate the performance of the marine blend cement formed using sodium silicate-based nanosilica with high resistance against chloride. Four types of binder containing the marine mixed cement were used, and, for each group, the percentage of binder to water ratio was also varied across three conditions, set to be 35, 40, and 45%. Consequently, a total of 12 batches were prepared for this investigation.

The detail of the binders applied in this experiment is listed in Table 1. The control mix “Mix A” used only ordinary Portland cement (OPC), whereas “Mix B” and “Mix C” were designed to test low heat cementitious material, as applied to reduce the high hydration heat that typically occurs in the mass of marine structures or infrastructure. In addition, the marine mixed cement “Mix D” that is the focus of the present paper was evaluated in terms of its suitability for use as a binder for marine structures by changing the mix ratio of “Mix B” and by mixing the SS (Table 1).

### 3.2. Materials

The density of OPC used in this experiment was 3150 kg/m^3^; those of the furnace slag powder and the fly ash were 2940 and 2200 kg/m^3^, respectively. Each of these materials was stirred through premixing before the main mixing, ensuring that all the mixing materials were uniformly distributed in the final cement. The physical and chemical properties of the premixed binders are listed in Tables 2 and 3, respectively. River sand with a density of 2550 kg/m^3^ and a maximum size of 5 mm and crushed aggregate with a density of 2620 kg/m^3^ and a maximum size of 25 mm were used as the aggregates.

### 3.3. Mix Design of Concrete

In this experiment, the unit quantity was fixed to 165 kg/m^3^ and the water to binder ratio was set to be 0.35, 0.40, and 0.45 across each condition. As such, this work assesses the impact on the applicability of each concrete mix according to the quantity of the unit binder as well as the kind of binder. The mixture proportions of the concrete used in this experiment are listed in Table 4.

### 3.4. Testing Method

#### 3.4.1. Concrete Mixing

A forced mixing type mixer was used, into which the coarse and fine aggregate and the premixing binder (cementitious materials) were inserted, after which dry mixing was conducted for 30 s. After this dry mixing, the water and superplasticizer were inserted and mixed for 60 s so as to be evenly released throughout the mixture.

#### 3.4.2. Compressive Strengths and Simple Heat of Hydration

**Figure 6:**
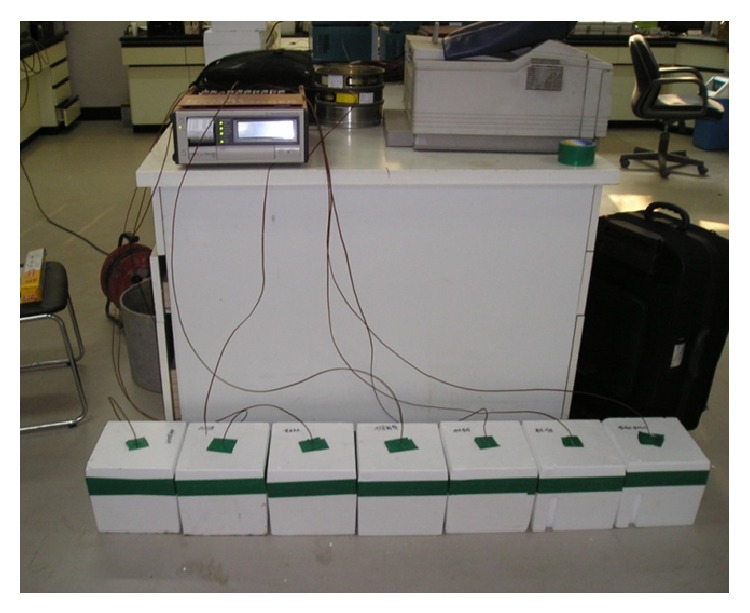
Simple hydration heat test.

For the compressive strength test mold, Ø100 × 200 mm cylinders were used, and demolding took place 24 h later. Curing was then carried out until the experiments were performed, in an environment set at 20°C.  Figure 6 is a photo of setting of a simple hydration heat test.

#### 3.4.3. Accelerated Carbonation and Sulfate Erosion Tests

Specimens of accelerated carbonation were produced in 100 × 100 × 400 mm cubic moulds. After 28 days, the two ends of each specimen were coated with epoxy resin to ensure that carbon dioxide (CO_2_) could diffuse only into the specimens in a two-dimensional model. The specimens were inserted into an accelerated carbonation tester so as to maintain a temperature of 20 ± 2°C, a relative moisture of 60 ± 5%, and CO_2_ concentration of 10 ± 0.5%. From this, we measure the depth of carbonation by using phenolphthalein solution at the ages of 7, 28, 56, and 91 days.

For a sulfate erosion test, after curing of 28 days, cylindrical specimens of Ø100 × 200 mm in size were submerged in sulfuric acid solution of 5%. Then, the weight loss of each specimen was measured at the ages of 7 and 28 days, respectively.

#### 3.4.4. Chloride Migration Coefficient

Evaluation to examine the penetration and the migration of chloride ions was performed, in line with this study's aim of developing high chloride-resistant concrete for the use in marine structures. The chloride resistance was examined through nonsteady state migration test, named NT build 492 [17, 18].

**Figure 7:**
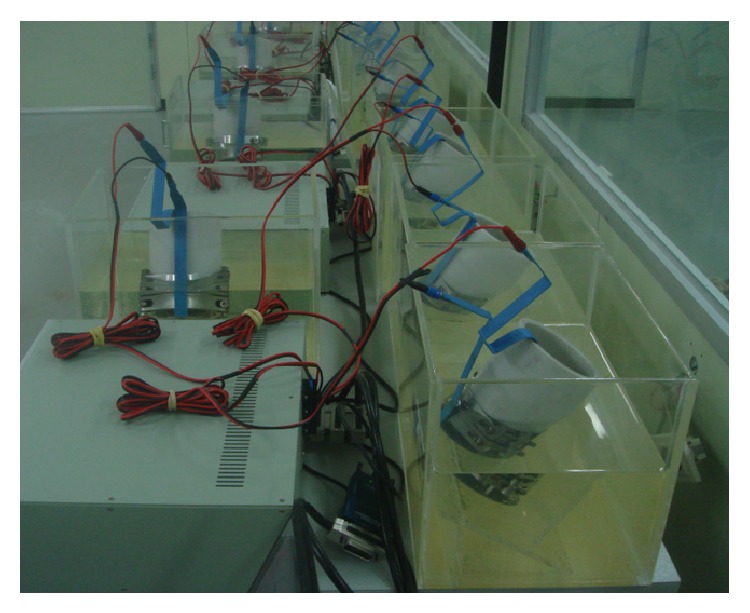
Chloride migration test.

Prior to testing, for each binder type cylindrical specimens of Ø100 × 200 mm were cut in half and specimens of 50 ± 2 mm thickness were then produced by cutting in half again. These specimens were prepared so that the side that was first cut was exposed to the chloride solution. The chloride-resistance test was performed by selecting the applied voltage level and test duration based on the current value when 30 v voltage was applied, where 0.3 N NaOH solution (approximately 12 g NaOH in 1 liter distilled water) was the positive pole and 10% NaCl solution (100 g NaCl in 900 g tap water) was the negative pole (Figure 7).

The penetration depth of the chloride was measured as the depth of discoloration of the specimen when spraying with 0.1 M AgNO_3_ solution by splitting (cutting in half) the specimen after completion of the test. The chloride migration coefficient could then be calculated in accordance with NT build 492 [17] on the basis of the measurement results for the thickness of the specimens, applied voltage, test duration, the penetration of chloride, and the temperature of the aqueous solution.

## 4. Result and Discussion

### 4.1. Compressive Strength

**Figure 8:**
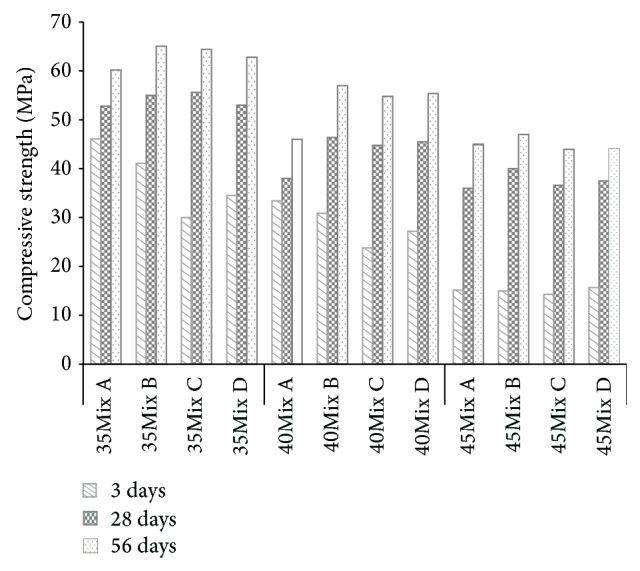
Compressive strength.

The binder type and the compression strength by age, as according to the W/B ratio, are shown in Figure 8. Until the seventh day, Mix A showed the highest compression strength, but compression strengths of similar levels in all mix types were shown due to a function of the cementitious material at the ages of 28 days and 56 days. The results of measuring mortar compressive strength in previous studies on SS have shown 41% and 82% higher compression than those for ethyl silicate-based nanosilica and silica fume, respectively, and these results have been understood in terms of the influence of quick pozzolanic reaction and pore-filling. However, it can be suggested that these advantages were not fully displayed here because the mixed quantity of SS was very small for the concrete compression strength, but it is analyzed that the mixing of SS is not the hazard elements in the development of strength.

### 4.2. Simple Heat of Hydration

**Figure 9:**
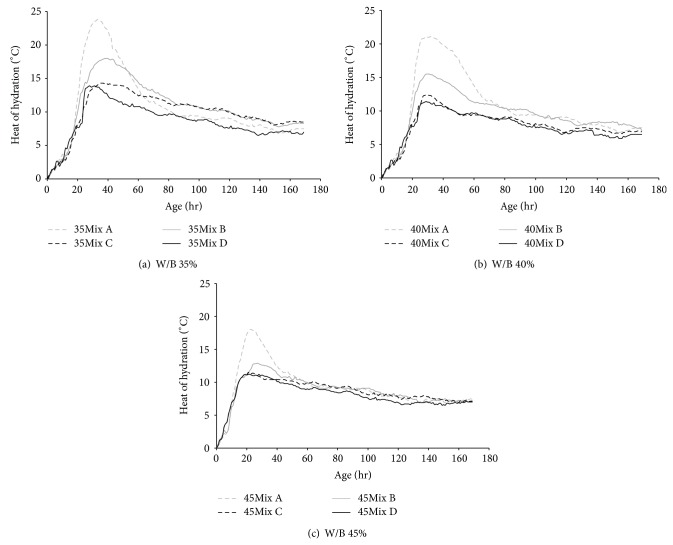
Hydration heat of concretes.

Figure 9 shows the results of hydration heat of specimens: Figures 9(a), 9(b), and 9(c) are the results of 0.35, 0.40, and 0.45 W/B specimens, respectively. The highest temperatures were observed in Mix A, and the lowest temperatures were shown in Mix D. At the peak point, the temperatures in Mix A were 24.2°C, 21.1°C, and 17.2°C as shown in Figures 9(a), 9(b), and 9(c), whereas corresponded temperatures of Mix D were 14.1°C, 11.4°C, and 11.0°C. The different temperature records in different mixes are mainly due to the different amounts of ordinary Portland cement (OPC). High temperature records generally observed in the specimens with low W/B are also the cause of high amounts of OPC used.

It is known that increasing the amount of OPC increases the heat of hydration. Partial replacement with mineral admixtures leads to low hydration heat as observed in Mix B, Mix C, and Mix D in Figure 9. Therefore, in marine environment where the large volume of concrete is normally placed, Mix C and Mix D that contain appropriate amounts of partial replacements with high W/B are recommended to reduce the hydration heat and to resist the thermal cracks.

### 4.3. Accelerated Carbonation and Sulfate Erosion Tests

**Figure 10:**
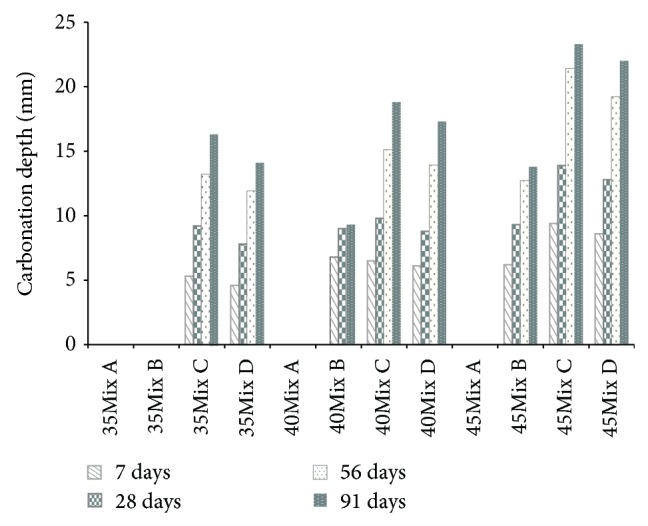
Carbonation depth.

Carbon dioxide and sulfate are the external harmful chemical species that deteriorate the durability of concrete [19].  Figure 10 shows the results of an accelerated neutralization test. The ingress of carbon dioxide determines the depth of carbonation of concrete specimens. Good resistance against the ingress of carbon dioxide was best achieved in Mix A. In Mix A, none of carbonations was proceeded in all specimens. Mix B was the next, followed by Mix D and Mix C. The worst results were found in Mix C.

In general, partial replacement with mineral admixtures such as fly ash and slag consumes the amount of Ca(OH)_2_, which in turn deteriorates the level of resistance against carbonation [20, 21]. Hence, for the concrete with high volume of partial replacement, it is important to note that the addition of pore-filling materials can be used as a second defence line to densify the microstructures of concrete, which resulted in resisting the ingress of harmful ionic species, such as carbon dioxide. As can be seen in Figure 10, although the mixture proportion of Mix C and Mix D was very similar and contained high volume of partial replacements, better results obtained from Mix D were attributed to the inclusion of SS that can densify the microstructures by filling the pores in concrete.

Sulfate attack becomes the main subject for the degradation of RC structures in marine environments involving chlorine ions [22]. Ettringite formation (made from an Al-bearing compound, gypsum, and tricalcium silicate) immersed in sulfate solution indicates the expansion phenomenon, as dependent on the elapse of time [23], and this phenomenon will ultimately reduce the service life of concrete structures [24].

**Figure 11:**
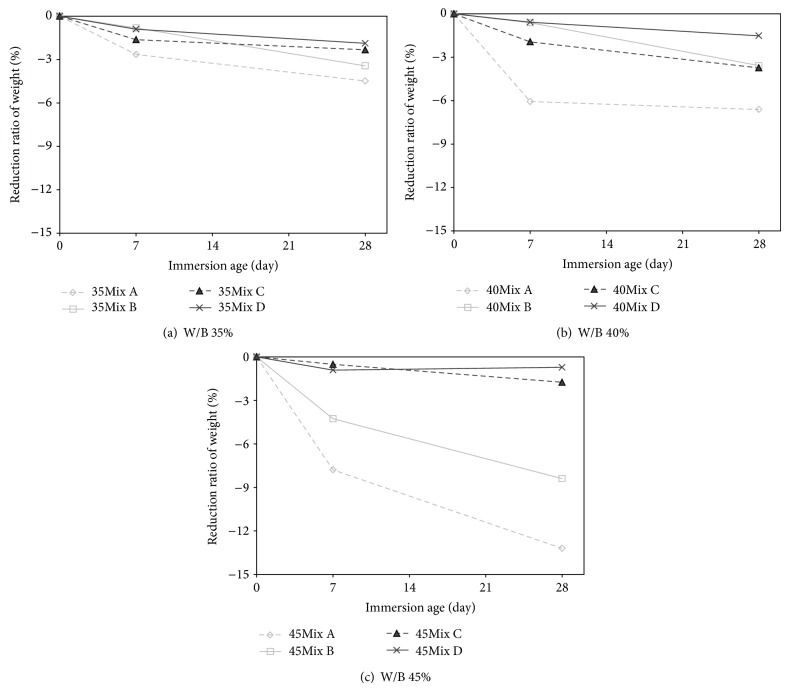
Weight loss of concretes due to sulfate attack.

Figure 11 shows the weight loss of the specimens submerged in the sulfate solution of 5% at room temperature for 28 days. The results of 0.35, 0.40, and 0.45 W/B specimens were separately plotted in Figures 11(a), 11(b), and 11(c). In Figure 11, Mix A is expressed with a gray dashed line and diamond symbols, Mix B is expressed with a gray solid line and rectangular symbols, Mix C is expressed with a black dashed line and triangle symbols, and Mix D is expressed with a black solid line and cross symbols. As expected, it was very clear that the lower the W/B ratio of each specimen is, the higher the level of sulfate resistance is; that is, for equivalent mixes, all specimens in Figure 11(a) had lower weight losses, compared to others shown in Figures 11(b) and 11(c).

It should be noted that, in marine structures, the W/B ratio of concrete placing in practice is mostly high, in order to reduce the heat of hydration. This is because the overall volume of the concrete applying to the marine structures is large. From the results observed in Figure 11, it can be said that the concrete in marine environment is exposed to the high risk of sulfate attack. Hence, to solve this problem, the development of a new concrete that can reduce the heat of hydration and at the same time can resist the sulfate attack is required.

This study found that the inclusion of sodium silicate-based nanosilica (SS) even under a high W/B condition (Figure 11(c)) can resist the sulfate attack. For example, as for Mix A without the inclusion of SS, the weight loss was 4.4% in Figure 11(a) (=W/B 0.35), 6.6% in Figure 11(b) (=W/B 0.40), and 13.2% in Figure 11(c) (=W/B 0.45), whereas, as for Mix D with the inclusion of SS, the results were 1.8% in Figure 11(a) (=W/B 0.35), 1.5% in Figure 11(b) (=W/B 0.40), and 0.7% in Figure 11(c) (=W/B 0.45). The increase of W/B did not deteriorate the Mix D specimen. This implies that the inclusion of SS in Mix D repels the ingress of sulfate ions.

In addition, in Figure 11, it can be seen that the partial replacement of cement with slag or fly ash was helpful in protecting the concrete from the sulfate attack, regardless of W/B. This effect is commonly reported in the open literatures. Better results observed in Mix B and Mix C as compared with Mix A in Figures 11(a), 11(b), and 11(c) were attributed to the fact that both the pozzolanic reaction in fly ash and the potential hydraulic reaction in slag consume Ca(OH)_2_ in the cementitious composites. Less amount of the Ca(OH)_2_ in Mix B and Mix C decreased the amounts of gypsum and ettringite formation, which is the cause of an expansive chemical reaction. The excellent result in Mix D is arising from the combined effect of the inclusion of SS and the partial replacement with fly ash and slag.

### 4.4. Chloride Migration Coefficient

**Figure 12:**
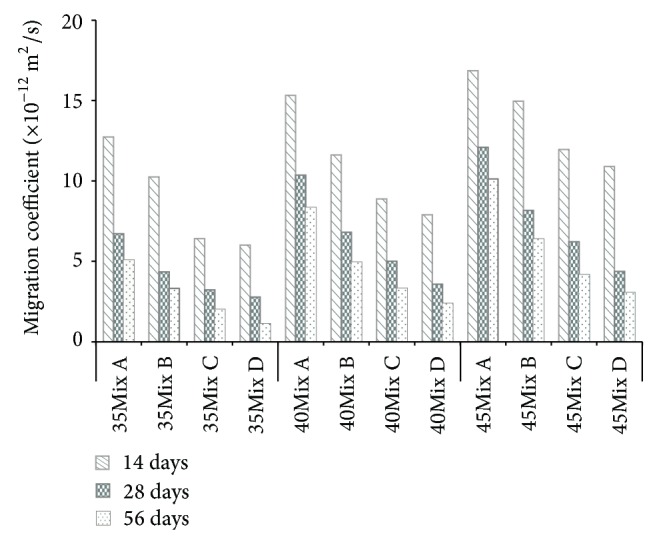
Chloride migration coefficient.

Figure 12 shows the chloride diffusion coefficients of all specimens tested by NT build 492. Overall, low W/B ratio decreased the chloride diffusion coefficients. This is because the diffusion of chloride ions is dependent on the microstructure of concrete, especially on the level of porosity. High volume of pores (especially connected pores) in concrete easily allows the ingress of the chloride ions. Interfacial transition zones and bleeding pores are the some of connected pores in concrete, and they are highly related to W/B ratio. The higher the W/B ratio is, the higher the volume of connected pores in concrete is, leading to the increase of the chloride diffusion.

In general, low W/B ratio increases the compressive strength of concrete. Hence, the reason for the good results in Mix C and Mix B as compared with Mix A in Figure 12 can be best explained with the strength results as previously discussed in Figure 8.

Most importantly, however, the parameter of the W/B cannot explain the results observed in Mix D, which has the best result in Figure 12. The highest resistance to chloride penetration in Mix D is attributed to the special surface treatment on SS with the inert halogen, as previously described in Figure 4. The electrical equilibrium state that is induced through the formation of the negative charge on the surface of SS is maintained, so that the diffusion of chloride ions can be prohibited.

**Figure 13:**
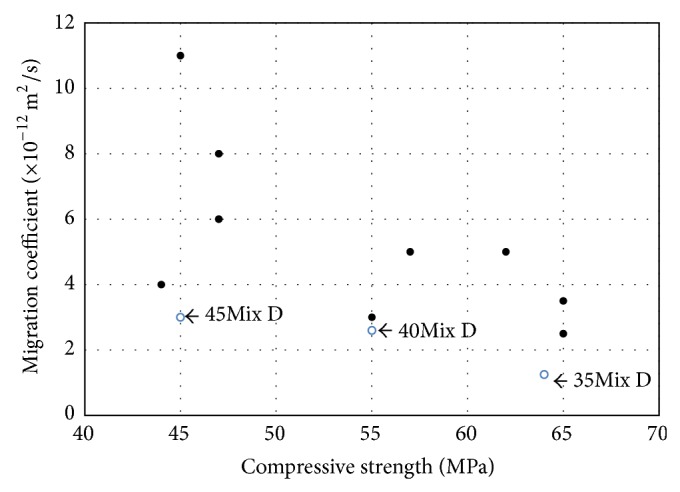
Strength versus chloride migration coefficient of all specimens tested at the age of 56 days.

In the literature, with similar test conditions, such as W/B ratio (0.35), test apparatus (NT build 492) with the voltage setting of 30, Teng et al. [25] investigated the effect of ultrafine slag and reported that the coefficients of chloride migration were ranged from 6.39 × 10^−12^ m^2^/s to 8.35 × 10^−12^ m^2^/s and Celik et al. [26] examined the effect of fly ash and found that the coefficients of chloride diffusion were ranged from 6.01 × 10^−12^ m^2^/s to 10.18 × 10^−12^ m^2^/s. These results are much higher than the results obtained from Mix D with SS inclusion, ranging between 1.11 × 10^−12^ and 7.89 × 10^−12^ m^2^/s (W/B 35% in Figure 12). The effectiveness of SS with the special surface treatment on the prevention of chloride attack is further discussed in Figure 13.

In Figure 13, the data obtained from a chloride diffusion test (Figure 12) is compared with the results of compressive strength (Figure 8). All data is from the representative samples tested at the age of 56 days. Again, it was very clear that Mix D had the lowest chloride diffusion coefficient. It should be noted that the effectiveness of SS was especially important on the sample with 0.40 W/B. This result (strength = 45 MPa) was over 3.5 times better than the worst result obtained from Mix A (strength = 45 MPa) at the equal strength level and was even similar to some of the samples with strengths of 65 MPa. Therefore, with SS inclusion, it is expected that the aforementioned problem of high W/B concrete structures in marine environment can be solved.

## 5. Conclusion

In order to increase resistance to the external chloride penetration of concrete structures in saline environments, a new sodium silicate-based nanosilica is used as partial replacement of cement. The following conclusion can be drawn from the experiments tested in this study.

- The compressive strength of Mix D with ordinary Portland cement (OPC) of 35%, blast furnace slag (BS) of 39.5%, fly ash (FA) of 25%, and sodium silicate-based nanosilica (SS) of 0.5% is similar to other concrete of Mix A (100% OPC), Mix B (60% OPC + 40% BS), and Mix C (40% OPC + 40% BS + 20% FA). Hence, the adverse effect of SS is not found in this test. This is probably due to that fact that the amount of SS inclusion is small.
- The heat of hydration testing indicates the differences in temperature based on the extent of the hydration reaction for OPC. It can be suggested that Mix C and Mix D have clear advantages for the reduction of temperature cracking because Mix A, Mix B, Mix C, and Mix D indicated 23.8°C, 18.0°C, 14.3°C, and 13.9°C, respectively, at both 35% W/B and the highest heat-generating temperature.
- In this study, three durability measuring items are used to assess the suitability of the proposed marine concrete, namely, carbonation, sulfate attack, and chloride migration. Among these, carbonation shows a particularly significant change according to the mixed amount of OPC in the binder, that is, the amount of cement substitute used. This variable is analyzed based on the results of the consumption Ca(OH)_2_ of cement hydration products, and the carbonation resistance is superior in the order of Mix A, Mix B, Mix D, and Mix C at most ages.
- In the sulfate attack results, which is another important tool for testing as applicable to marine environments, the item for which the resistance showed the lowest (reduced) value is the Mix A with a higher W/B ratio. However, if Mix D is applied as the binder, the sample shows superior erosion resistances of 5.1%, 11.5%, and 13.2%, at W/B ratios of 35, 40, and 45%, respectively, compared to the results for the Mix A.
- The resistance characteristics for chloride diffusion are superior in the order of Mix D, Mix C, Mix B, and Mix A. This performance difference is shown proportionally, and it is clear that Mix D shows the strongest diffusion coefficient across all W/B ratios.

## Figures and Tables

**Table 1:** OPC and partial replacement ratio in each mix.

Abbreviation	Proportion of binders (%)
Mix A	OPC^a^ (100)
Mix B	OPC (60) + BS^b^ (40)
Mix C	OPC (40) + BS (40) + FA^c^ (20)
Mix D	OPC (35) + BS (39.5) + FA (25) + SS^d^ (0.5)

^a^Ordinary Portland cement.

^b^Ground granulated blast furnace slag.

^c^Fly ash.

^d^Sodium silicate-based nanosilica.

**Table 2:** Physical properties of premixed binders.

Abbreviation	Density (kg/m^3^)	Fineness (m^2^/kg)	Setting time (min)		Compression strength (MPa)		
			Initial	Final	3 days	7 days	28 days
Mix A	3150	337	220	400	20.2	27.0	35.8
Mix B	3120	402	185	425	18.1	28.7	48.7
Mix C	2810	373	265	510	11.2	21.0	35.8
Mix D	2800	446	250	555	12.5	22.4	35.7

**Table 3:** Chemical composition of premixed binders.

Abbreviation	Chemical composition (%)						
	SiO_2_	Al_2_O_3_	Fe_2_O_3_	CaO	MgO	SO_3_	Ignition loss
Mix A	20.83	5.30	3.00	62.39	2.31	2.30	2.44
Mix B	26.61	9.88	1.63	53.33	3.66	2.19	0.80
Mix C	30.23	15.75	4.05	44.01	3.48	1.93	1.27
Mix D	28.14	15.87	3.06	45.95	4.98	4.12	0.65

**Table 4:** Design of concrete mix.

No.	W/B (%)	S/a (%)^a^	W (kg/m^3^)	Unit volume weight (kg/m^3^)			Abbreviation
				B^b^	S^c^	G^d^	
1	35	44.0	165	471	747	980	35 Mix A
2		43.5			733	981	35 Mix B
3		42.5			701	977	35 Mix C
4		42.5			702	979	35 Mix D

5	40	45.5	165	413	794	981	40 Mix A
6		45.0			781	984	40 Mix B
7		44.0			749	983	40 Mix C
8		44.0			751	985	40 Mix D

9	45	46.5	165	367	829	984	45 Mix A
10		46.5			825	978	45 Mix B
11		46.5			812	963	45 Mix C
12		46.5			813	964	45 Mix D

^a^Sand to aggregate ratio.

^b^Premixed binder (Table 1).

^c^Sand.

^d^Gravel.
